# Causal associations between gut microbiota and primary biliary cholangitis: a bidirectional two-sample Mendelian randomization study

**DOI:** 10.3389/fmicb.2023.1273024

**Published:** 2023-11-15

**Authors:** Jiahao Zhang, Gefeng Wu, Yuhong Tang, Huanxiang Liu, Xinyu Ge, Rui Peng, Jun Cao, Daoyuan Tu, Bingbing Su, Shengjie Jin, Guoqing Jiang, Chi Zhang, Dousheng Bai

**Affiliations:** ^1^Department of Hepatobiliary Surgery, Clinical Medical College, Yangzhou University, Yangzhou, Jiangsu, China; ^2^The Yangzhou School of Clinical Medicine of Dalian Medical University, Dalian, Liaoning, China

**Keywords:** gut microbiota, primary biliary cholangitis, Mendelian randomization study, genome-wide association study, causal effect

## Abstract

**Background:**

Previous studies have suggested an association between gut microbiota and primary biliary cholangitis (PBC). Nonetheless, the causal relationship between gut microbiota and PBC risk remains unclear.

**Methods:**

A bidirectional two-sample Mendelian Randomization (MR) study was employed using summary statistical data for gut microbiota and PBC from the MiBioGen consortium and Genome-Wide Association Studies (GWAS) database to investigate causal relationships between 211 gut microbiota and PBC risk. Inverse variance weighted (IVW) method was the primary analytical approach to assess causality, and the pleiotropy and heterogeneity tests were employed to verify the robustness of the findings. Additionally, we performed reverse MR analyses to investigate the possibility of the reverse causal association.

**Results:**

The IVW method identified five gut microbiota that demonstrated associations with the risk of PBC. Order *Selenomonadales* [odds ratio (OR) 2.13, 95% confidence interval (CI) 1.10–4.14, *p* = 0.03], Order *Bifidobacteriales* (OR 1.58, 95% CI 1.07–2.33, *p* = 0.02), and Genus *Lachnospiraceae_UCG_004* (OR 1.64, 95%CI 1.06–2.55, *p* = 0.03) were correlated with a higher risk of PBC, while Family *Peptostreptococcaceae* (OR 0.65, 95%CI 0.43–0.98, *p* = 0.04) and Family *Ruminococcaceae* (OR 0.33, 95%CI 0.15–0.72, *p* = 0.01) had a protective effect on PBC. The reverse MR analysis demonstrated no statistically significant relationship between PBC and these five specific gut microbial taxa.

**Conclusion:**

This study revealed that there was a causal relationship between specific gut microbiota taxa and PBC, which may provide novel perspectives and a theoretical basis for the clinical prevention, diagnosis, and treatment of PBC.

## 1. Introduction

Primary biliary cholangitis (PBC) is an autoimmune liver disease characterized by chronic cholestasis, fibrosis, and the destruction of small intrahepatic bile ducts, leading to the development of irreversible cirrhosis and liver failure (Levy et al., 2023). A comprehensive review of epidemiological investigations revealed that the incidence of PBC varies between 0.3 and 5.8 per 1,000 individuals, with a discernible upward trend in prevalence rates over time (Lu et al., 2018). Despite being rare, PBC imposes a weighty clinical burden compared to its incidence and prevalence in the population. Regrettably, the etiology of PBC remains largely unresolved due to the complex interaction between environmental triggers and genetic susceptibility factors (Lleo et al., 2020).

The human gut microbiome, comprised of a diverse array of microorganisms, performs a vital function in metabolic processes, immune regulation, and the preservation of gut integrity (Hou et al., 2022). An increasing body of evidence indicates that imbalances in the gut microbiota may play a role in the onset and advancement of PBC (Zhang L. et al., 2023). This connection arises from the linkage between the liver and intestine through the portal vein, forming a gut-liver axis (Wang et al., 2021). In a study, it was observed that the gut microbiota compositions in individuals with PBC significantly diverged from those in healthy controls. This disparity manifested as a decrease in several potentially advantageous microbiota, such as *Faecalibacterium* and *Ruminococcaceae*, along with an increase in opportunistic pathogens, including *Serratia* and *Yersiniaceae* (Zhou et al., 2023). Tang et al. also found that significant differences in the serum and fecal bile acid profiles between PBC patients without ursodeoxycholic acid (UDCA) treatment and the control group (Tang et al., 2018). Furthermore, an observational study indicated that the cholestasis in PBC patients may be related to impaired bile acid metabolism caused by the dysregulation of the gut microbiota. For instance, secondary bile acids (SBAs) were found to be inversely correlated with genera that were enriched in PBC (*Veillonella*, *Klebsiella*) and positively correlated with genera enriched in controls (*Bacillus*, *Oscillatory*) (Chen et al., 2020). In addition, the gut microbiota can also be involved in the development of PBC by increasing gut permeability, influencing the intestinal mucosal immune balance, and inducing abnormal immune activation (Harada et al., 2009; Allam-Ndoul et al., 2020; Furukawa et al., 2020). However, most existing research predominantly utilizes observational study designs, often featuring limited sample sizes, and may be influenced by confounding factors, such as lifestyle, environment, and age. While these discoveries have established a connection between gut microbiota and PBC, they have not definitively uncovered a specific cause-and-effect relationship.

Mendelian randomization (MR) is a novel statistical approach that facilitates the assessment of causality, similar to a randomized controlled trial, by leveraging the random assignment of genetic variants during conception (Birney, 2021). By employing single nucleotide polymorphisms (SNPs) as instrumental variables (IVs), MR enables the modeling and inference of causal effects, effectively mitigating the impact of confounding variables (Richmond and Davey Smith, 2022). Moreover, the non-reversibility of heredity in MR analysis is advantageous in addressing concerns related to reverse causation interference (Xu Q. et al., 2022). Although the MR method has been employed in several studies to examine the potential causal relationship between gut microbiota and different diseases (Li et al., 2022; Liu et al., 2023; Long et al., 2023), there is currently limited evidence regarding the causal association between gut microbiota and PBC.

In this study, we performed the first bidirectional two-sample MR analysis on summary data from Genome-Wide Association Studies (GWAS), investigating the causal relationship between gut microbiota and PBC risk. Our findings highlight the causal influence of gut microbiota on PBC and identify specific gut microbiota markers that could provide value in diagnosing and treating PBC.

## 2. Methods

### 2.1. Study design

**Figure 1:**
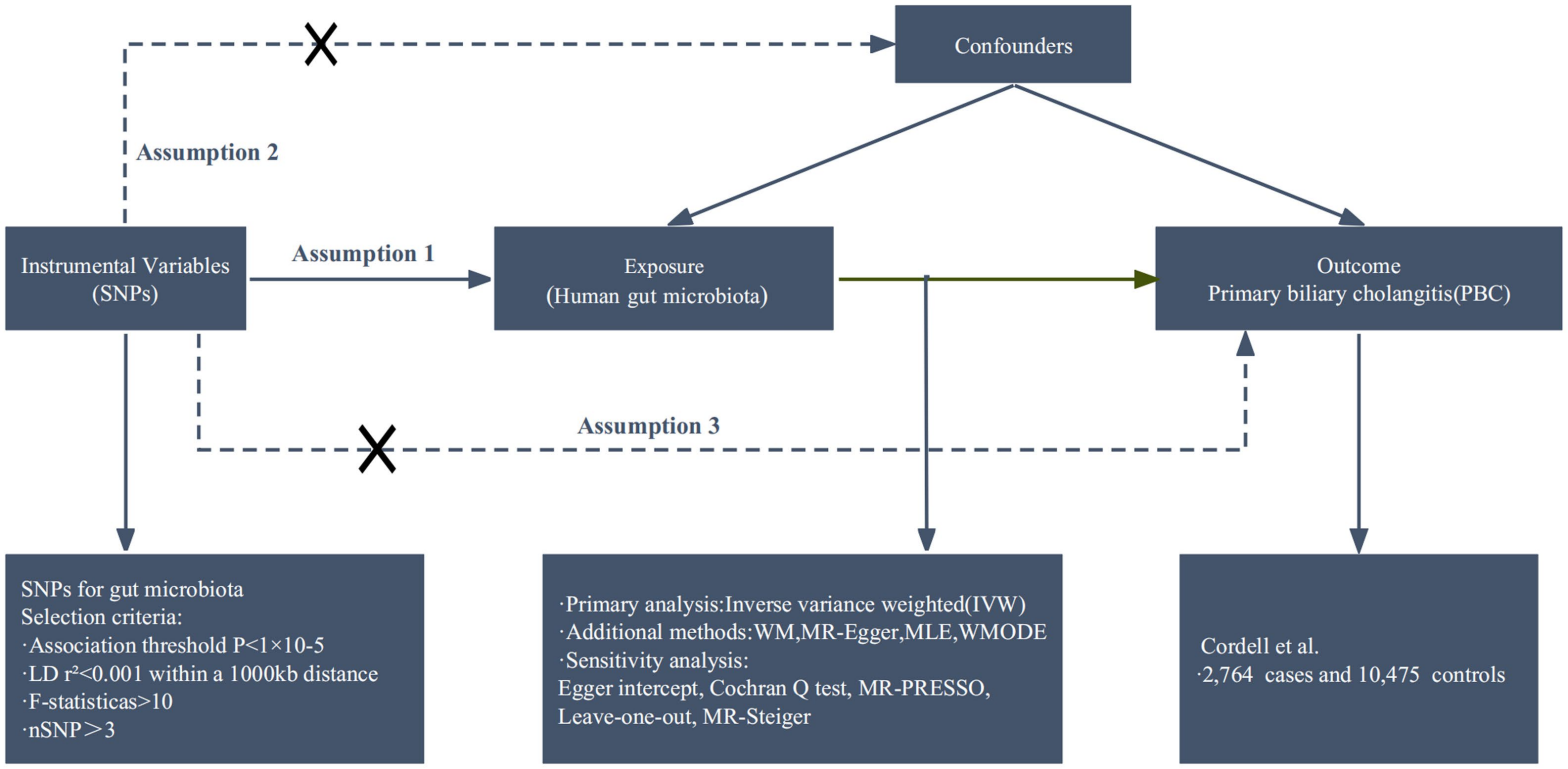
Overview of the Mendelian randomization analysis and three main assumptions. The workflow of the Mendelian randomization study exhibits causality between gut microbiota and PBC. Assumption 1 is a significant association between genetic variation and exposure; Assumption 2, there is no correlation between genetic variation and confounding factors; Assumption 3, genetic variants exert effects on the outcomes by influencing the exposure of interest. The arrows denote causal relations between two variables, pointing from the cause to the effect. The causal pathway is blocked if “X” is placed in the arrowed line. SNPs, single nucleotide polymorphisms; LD, linkage disequilibrium; WM, weighted median; WMODE, weighted mode.

We conducted a bidirectional two-sample MR analysis to investigate the potential correlation between gut microbiota and PBC. To mitigate the impact of confounding variables on the outcomes, the MR should meet three fundamental assumptions (Davies et al., 2018): (1) there must be significant associations between IVs and gut microbiota; (2) IVs should not be correlated with confounding factors that are unrelated to gut microbiota; (3) the impact of IVs on PBC should be solely mediated through gut microbiota (Figure 1).

### 2.2. Gut microbiota data sources

The genetic information of gut microbiota was acquired from the largest GWAS conducted by the MiBioGen consortium, which encompassed 5,717,754 SNPs and 18,340 participants of 16S rRNA gene sequencing data from 24 cohorts (Kurilshikov et al., 2021). The study included a predominantly European ancestry, with 16 cohorts and 13,266 samples. The researchers identified a comprehensive set of 211 gut microbiomes, covering the species to the genus level, comprising 9 phyla, 16 classes, 20 orders, 35 families, and 131 genera. The cohort dataset employed in this study underwent meticulous adjustments for sex, age, genotyping batch, and the first ten principal components, as conducted by the original investigators of the respective cohorts (Kurilshikov et al., 2021). Fifteen microbial taxa were removed without specific species names (13 unknown families and 2 unknown genera). A total of 196 microbial taxa were included for analysis.

### 2.3. The data source for PBC

The summary statistic of PBC was extracted from the genome-wide meta-analysis of Cordell et al., including 2,764 cases and 10,475 controls of European ancestry (Cordell et al., 2021). All cases of PBC included in the cohorts met the diagnostic criteria specified by the European Association for the Study of the Liver (EASL).

### 2.4. Selection of instruments variables

To ensure the accuracy of the results, a rigorous data screening process was conducted on the extracted information from MiBioGen. Considering the limited number of gene loci that achieve genome-wide significance levels in GWAS for the gut microbiota (*p* < 5 × 10^−8^), we chose to use exposure data with a threshold of *p* < 1 × 10^−5^ to broaden the scope of correlation findings (Sanna et al., 2019). To ensure independence among the SNPs, a linkage disequilibrium (LD, R^2^ > 0.001 and within 10,000 kb) was conducted based on the European-based 1,000 Genomes Project (The 1000 Genomes Project Consortium, 2010). Following the principle that the selected SNPs affect exposure and outcome through identical alleles, we removed palindromic SNPs without A/T or C/G polymorphisms from the IVs (Zhang et al., 2021).

The impact of each SNP on gut microbiota was analyzed by assessing the F and R^2^  values utilizing the specified formula: F = [R^2^ × (N-2)]/(1- R^2^), R^2^ = [2 × β^2^ × EAF × (1-EAF)]/[2 × β^2^ × EAF × (1-EAF) + 2 × SE^2^ × N × EAF × (1-EAF)] (Burgess et al., 2011; Levin et al., 2020). It is worth noting that N and EAF in these formulas indicate the sample size and effect allele frequency, respectively. Additionally, the SNP’s impact size and standard error on gut microbiota are represented by β and SE. We excluded SNPs with an F statistic of less than 10, as these SNPs lacked adequate validity in the analyses. Furthermore, gut microbiota with fewer than 3 correlated SNPs across the genome was removed, following the requirement of having a minimum of 3 SNPs correlated with the exposure in specific sensitivity analyses using MR (Hemani et al., 2018).

### 2.5. Mendelian randomization analysis

In this study, the primary approach used to evaluate the causal relationship between gut microbiota and PBC without considering horizontal pleiotropy was the Inverse Variance Weighted (IVW) method (Burgess et al., 2013). Furthermore, to enhance the robustness of our findings, we utilized supplementary methodologies in conjunction with the IVW approach, which included MR-Egger, the weighted median (WM), the maximum likelihood estimator (MLE), and the weighted mode (WMODE). The supplementary methods should conform to the assumptions of their respective models. The WM method assumes that at least half of the SNPs are unaffected by pleiotropy (Bowden et al., 2016). However, even if more than 50% of the SNPs are affected by pleiotropy, the MR-Egger inference results will remain robust (Bowden et al., 2015). The MLE, or Maximum Likelihood Estimation, is a theoretical method used for point estimation in this study. Compared to the IVW method, the MLE method exhibits a lower standard error and yields unbiased results without heterogeneity or horizontal polymorphism (EPIC- InterAct Consortium et al., 2015). The weighted mode method is versatile in handling genetic variables challenging the pleiotropy hypothesis (Hartwig et al., 2017). The IVW approach was utilized as the primary foundation of this study, supplemented by four additional methods to strengthen and enhance the findings.

### 2.6. Sensitivity analysis

To enhance the credibility and robustness of our results, we performed a comprehensive series of sensitivity analyses. Two methods were utilized to scrutinize the horizontal pleiotropy: the MR-PRESSO global test and the MR Egger intercept test. A *p* value greater than 0.05 in both tests showed the absence of horizontal pleiotropy (Verbanck et al., 2018). To assess heterogeneity in this MR analysis, we utilized Cochran’s Q statistic (MR-IVW) and Rucker’s Q statistic (MR Egger). *p* values exceeding 0.05 indicated no significant heterogeneity (Hemani et al., 2018). Furthermore, we employed a leave-one-out sensitivity test to evaluate the potential impact of individual SNPs on causal associations (Xiang et al., 2021). To validate the second MR assumption, we conducted a comprehensive search in the PhenoScannerV2 database, exploring each IV and its corresponding proxy features (Kamat et al., 2019). Then, we subsequently excluded SNPs associated with potential confounders or risk factors (including inflammatory bowel disease, vitamin D concentrations, and obesity) (Xu H. et al., 2022; Zhang H. et al., 2023). Moreover, we applied the MR Steiger directionality test to assess the association between exposure and outcome (Hemani et al., 2017).

### 2.7. Reverse Mendelian randomization

In addition, we utilized a reverse MR analysis to evaluate the potential reverse causal association between PBC and gut microbiota. In this context, PBC was regarded as the exposure, and we extracted SNPs associated with PBC as the IVs (*p* < 5 × 10^−8^). Similar to the forward MR, a selection process was conducted, which involved eliminating linkage disequilibrium and instrumental variables with an F statistic below 10. Significant gut microbiota identified from the forward MR analysis was then utilized as the outcome. Subsequently, a two-sample MR analysis was utilized to determine the causal association between PBC and gut microbiota.

### 2.8. Statistical analysis

We established a robust causal association between gut microbiota and PBC based on the following criteria (Wang et al., 2023): (1) data analysis using the IVW method showed a statistically significant difference (*p* < 0.05); (2) consistent estimates were obtained from all five methods; (3) the Cochran’s Q test, MR-Egger test, and MR-PRESSO global test yielded non-significant results (*p* > 0.05); and (4) the MR Steiger directionality tests confirmed the concordance of causal direction. The above analyses were conducted using the Two-Sample-MR package (version 0.5.7) within the R software environment (version 4.2.3).

### 2.9. Ethics statement

Every GWAS incorporated in this study is publicly accessible via the original research publications and has obtained ethical approval from their respective institutions.

## 3. Results

### 3.1. Instrumental variable selection

We conducted quality control and identified 995 SNPs associated with PBC (Supplementary Table S1), which involved 171 gut microbiota taxa (including 109 genera, 29 families, 19 orders, 15 classes, and 7 species). The F statistics for IVs range from 16.91 to 88.42, showing the absence of weak IVs bias. The essential data for all IVs are presented in Supplementary Table S1. It’s important to highlight that in cases where two microbiota genera shared the same SNPs in our study, we opted for taxonomically distinct options (e.g., we selected the Order *Selenomonadales* instead of the Class *Negativicutes*, and the Order *Bifidobacteriales* instead of the Family *Bifidobacteriaceae*) (Ye et al., 2023).

### 3.2. Causal associations of gut microbiota with PBC

**Figure 2:**
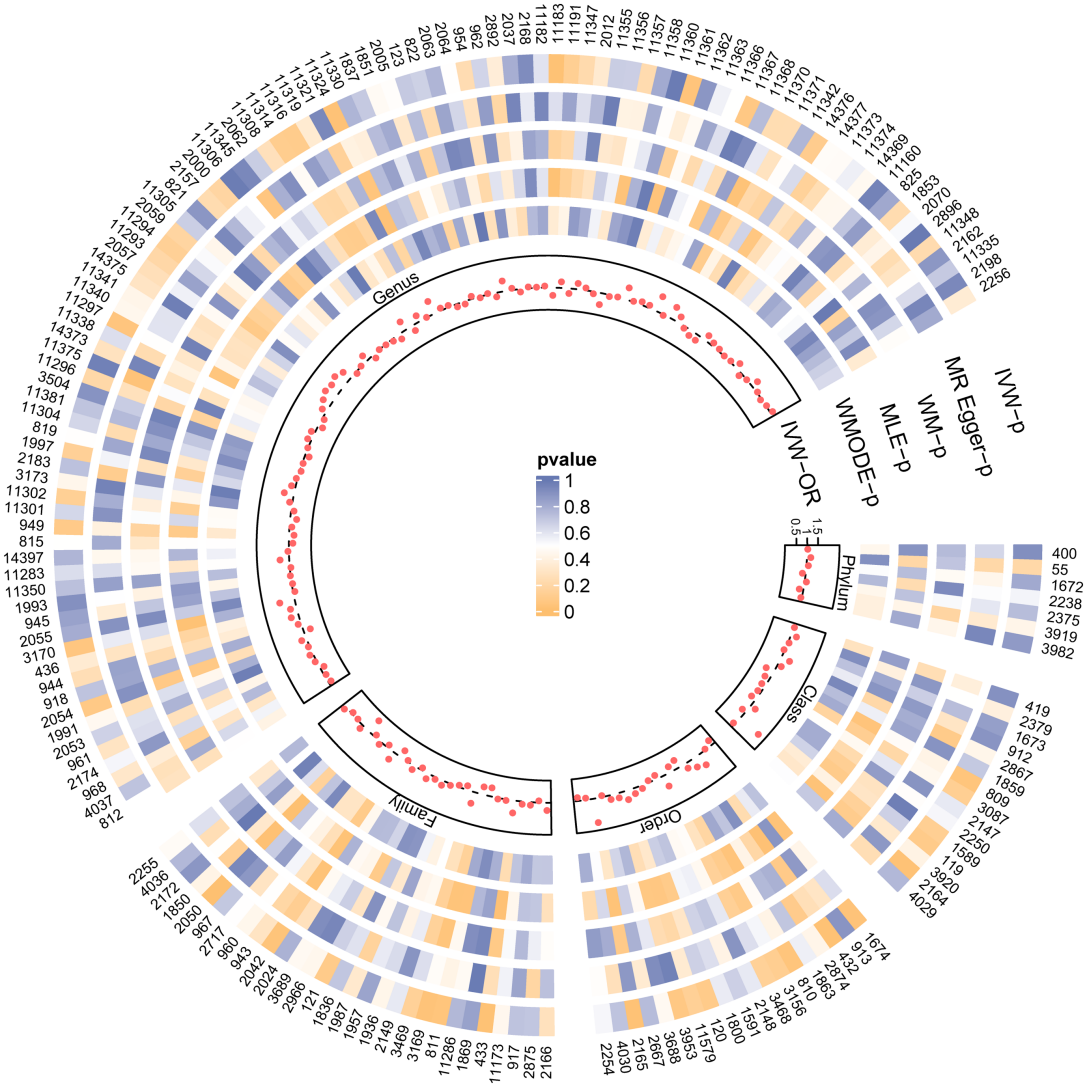
SNPs influence the causal effect with five MR methods. Each red dot represents the causal effect on PBC of each SNP with IVW, and each region corresponds to a different level of gut microbiota, including phylum, class, order, family, and genus. The gray dashed line represents OR = 1. Circles from outside are the *p*-value of IVW, the p-value for MR-Egger, the p-value for WM, the p-value for MLE, and the p-value for WMODE. The outermost circle is each gut microbiota’s ID, corresponding to the bacterial taxon name in Supplementary Table S1. OR, odds ratio; IVW, inverse-variance-weighted estimate; WM, weighted median; MLE, maximum likelihood estimator; WMODE, weighted mode.

**Figure 3:**
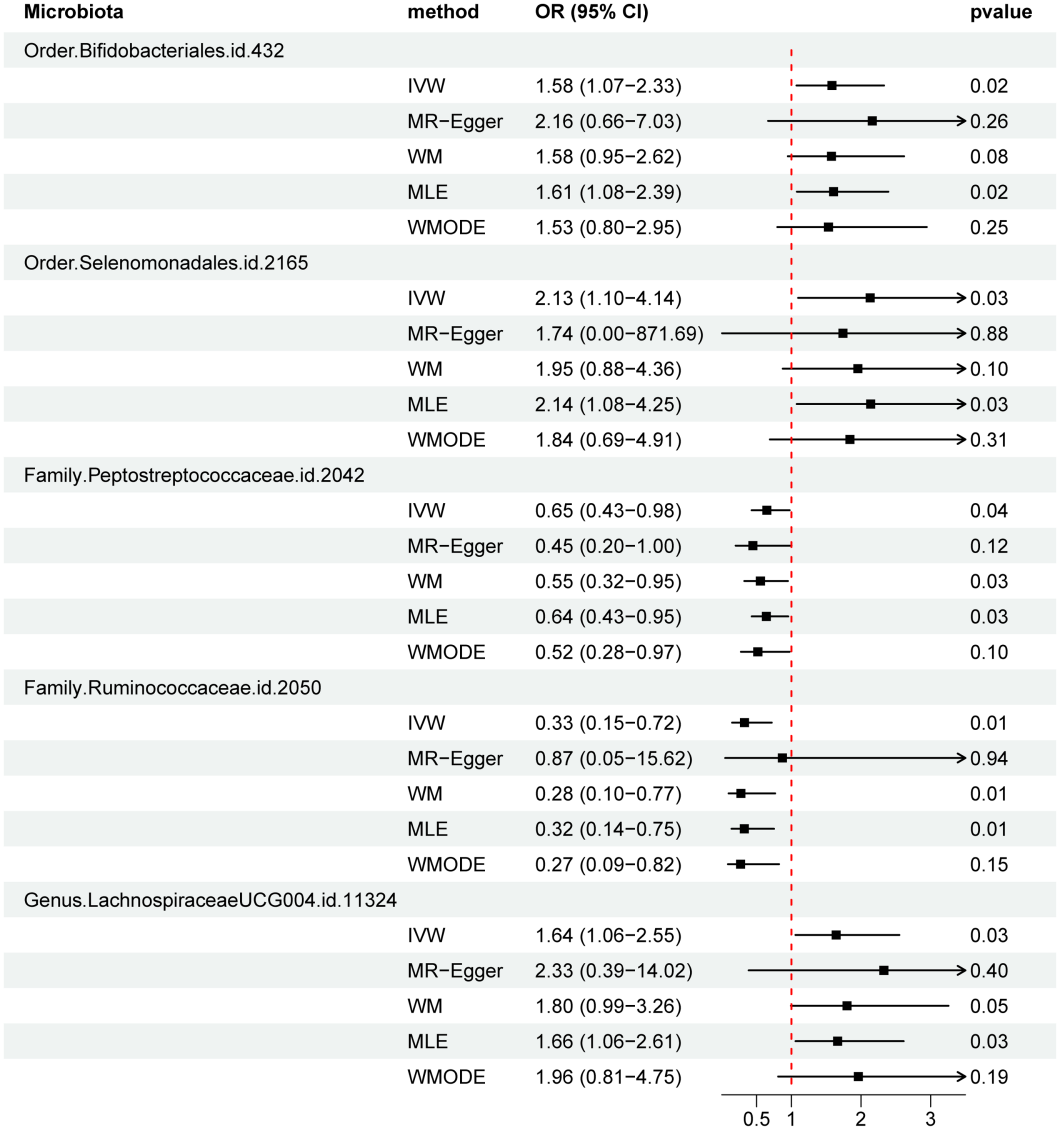
Forest plot of MR estimate for the association between gut microbiota and PBC. Lines signify 95% confidence intervals and are truncated where they exceed the plotted range (arrowheads). OR, odds ratio; IVW, inverse-variance-weighted; WM, weighted median; MLE, maximum likelihood estimator; WMODE, weighted mode.

Figure 2 and Supplementary Table S2 visually depict the initial findings on the connections between genetically represented gut microbiota and the risks associated with PBC. By applying a rigorous significance threshold (*p* < 0.05) using the IVW method and considering consistent directions across all five methods, we have discovered five gut microbiota taxa that demonstrate causal associations with PBC risk (Figure 3). The IVW analysis revealed a positive correlation between three gut microbiota taxa and PBC risk: Order *Selenomonadales* (OR 2.13, 95%CI 1.10–4.14, *p* = 0.03), Order *Bifidobacteriales* (OR 1.58, 95% CI 1.07–2.33, *p* = 0.02), and Genus *Lachnospiraceae_UCG_004* (OR 1.64, 95%CI 1.06–2.55, *p* = 0.03). These findings suggest a possible link between these specific gut microbiota taxa and an increased risk of PBC. In contrast, our analysis discovered a significant negative relationship between two gut microbiota taxa and the risk of PBC: Family *Peptostreptococcaceae* (OR 0.65, 95%CI 0.43–0.98, *p* = 0.04), and Family *Ruminococcaceae* (OR 0.33, 95%CI 0.15–0.72, *p* = 0.01). These findings suggest that the two gut microbiota taxa have a protective effect against the development of PBC. The results obtained from other complementary analytical methods consistently corroborated the findings of the primary analysis, thereby bolstering confidence in the genuine causal relationship. Although none of the MR findings reached the threshold for significance after applying Bonferroni correction for various testing, there were several *p*-values less than 0.05, which indicated nominal significance.

### 3.3. Sensitivity analyses and detection of pleiotropy

**Figure 4:**
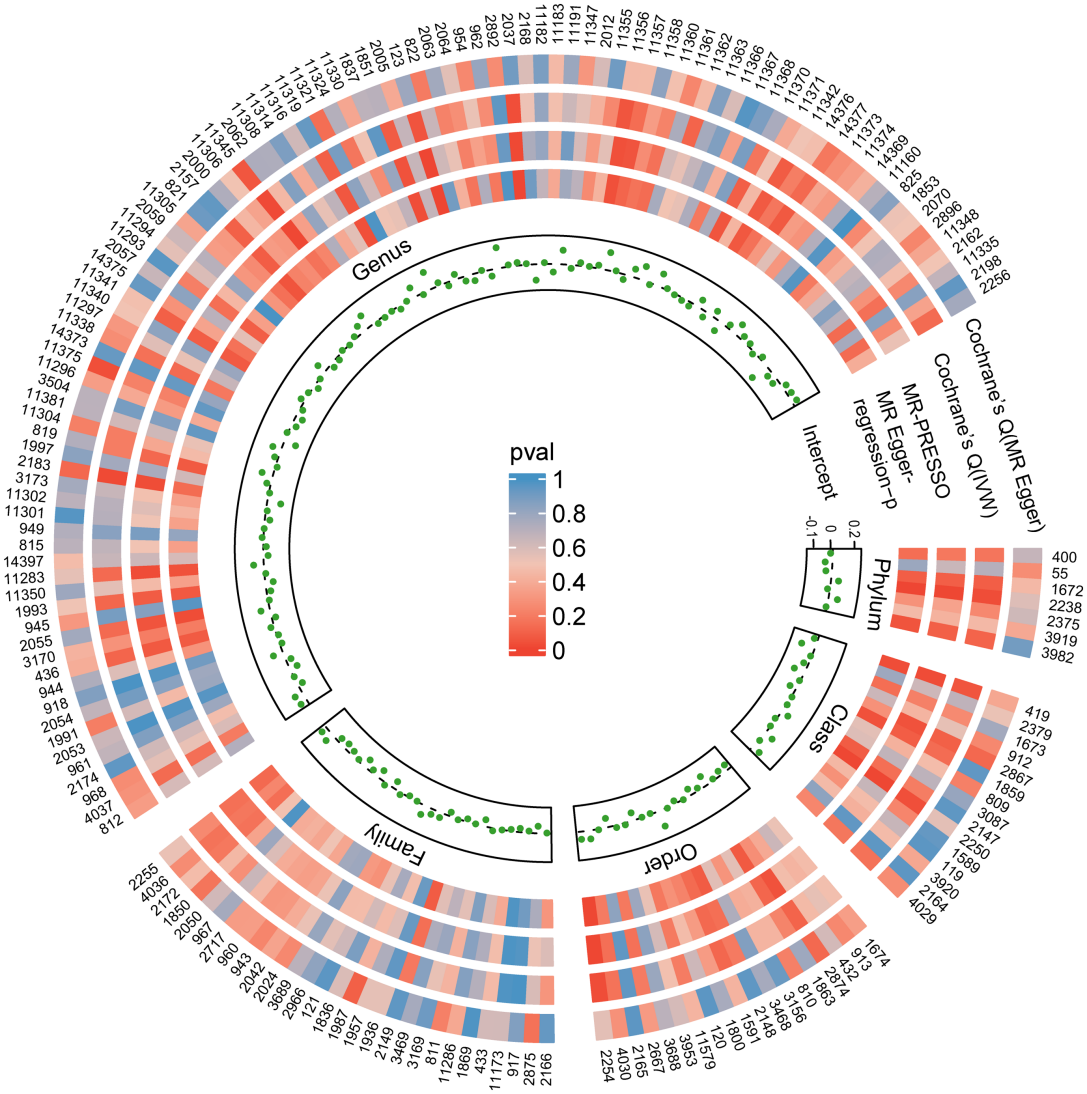
Sensitivity analysis results of all SNPs. Each green dot represents the intercept of MR-Egger, and each region corresponds to a different level of gut microbiota, including phylum, class, order, family, and genus. The gray dashed line represents intercept =0. Circles from outside are the *p*-values for Cochrane’s Q (MR-Egger), Cochrane’s Q (IVW), and the p-values for the MR-PRESSO, the p-values for the MR-Egger regression. The outermost circle is each gut microbiota’s ID, corresponding to the bacterial taxon name in Supplementary Table S1. IVW, inverse-variance-weighted estimate; MR, Mendelian randomization. MR-PRESSO, MR-Pleiotropy RESidual Sum and Outlier.

The results from all sensitivity assessments are aggregated and presented in Figure 4. Despite the varied results, Cochran’s Q tests indicate no significant heterogeneity among the IVs (*p* > 0.05). Furthermore, the MR-Egger intercept tests do not reveal any evidence of horizontal pleiotropy across the five gut microbiota taxa. Additionally, the subsequent MR-PRESSO analysis does not detect any significant outliers (Table 1). The scatter plots (Supplementary Figure S1) and leave-one-out plots (Supplementary Figure S2) do not exhibit any potential outliers in the IVs. Furthermore, after querying the SNPs as mentioned above for positive associations in PhenoScannerV2, we identified that only rs12894272 in the IVs of *Lachnospiraceae_UCG_004* was significantly associated with body mass index (BMI) (Supplementary Table S3). After removing the SNP, the causal effects of *Lachnospiraceae_UCG_004* remained significant (Supplementary Table S4). Moreover, the MR Steiger directionality test unequivocally confirmed the robustness of the five causal effects, indicating a consistent direction from the gut microbiota to PBC (Table 1). These findings suggest that the previously mentioned gut microbiota influences the observed causal associations between gut microbiota and PBC.

**Table 1:** Sensitivity analyses of MR results between gut microbiota and PBC.

Classification	Gut microbiota	Pleiotropy, *P*		Heterogeneity (Cochrane’s Q), *P*		Correct Causual direction	Steiger pval
		MR-Egger intercept	MR-PRESSO	IVW	MR-Egger		
order	Bifidobacteriales.id.432	0.61	0.45	0.38	0.47	TRUE	4.07E-15
order	Selenomonadales.id.2165	0.95	0.96	0.95	0.83	TRUE	3.26E-06
family	Peptostreptococcaceae.id.2042	0.36	0.39	0.36	0.37	TRUE	7.94E-09
family	Ruminococcaceae.id.2050	0.61	0.29	0.79	0.98	TRUE	7.19E-04
genus	Lachnospiraceae_UCG_004.id.11324	0.73	0.21	0.61	0.5	TRUE	3.61E-10

### 3.4. Reverse Mendelian randomization

A reverse MR analysis was utilized through the IVW to explore the potential causal association between PBC and five specific gut microbiota taxa. This study evaluates whether genetically predicted PBC could causally influence gut microbiota composition. After eliminating linkage disequilibrium, we identified 56 SNPs, each exhibiting a solid association with PBC and having an *F* value greater than 10 (Supplementary Table S5). The data presented in Supplementary Table S6 did not show any significant reverse causal association between PBC and gut microbiota taxa, including Order *Selenomonadales* (*p* = 0.09), Order *Bifidobacteriales* (*p* = 0.61), Family *Peptostreptococcaceae* (*p* = 0.25), Family *Ruminococcaceae* (*p* = 0.25), and Genus *Lachnospiraceae_UCG_004* (*p* = 0.71). The MR-Egger regression analysis showed that the *p*-values for the horizontal pleiotropy results of the *Selenomonadales* were less than 0.05. However, the MR-PRESSO test results showed no evidence of horizontal pleiotropy or outlier values, warranting further discussion (Supplementary Table S6). Moreover, no horizontal pleiotropy was detected in the remaining analyzed gut microbiota. The MR Steiger directionality tests consistently showed that PBC had a statistically significant influence on five gut microbiota (Supplementary Table S6).

## 4. Discussion

Using summary statistics from a large-scale GWAS, we employed a bidirectional two-sample MR approach to investigate the causal relationship between gut microbiota and PBC risk. Our findings provide compelling evidence indicating a negative correlation between an increased abundance of *Ruminococcaceae* and *Peptostreptococcaceae* and the risk of PBC. Conversely, *Selenomonadales*, *Bifidobacteriales*, and *Lachnospiraceae_UCG_004* may pose risk factors for PBC. Furthermore, the bidirectional MR analyses did not reveal any evidence of a reverse causal relationship. These discoveries offer valuable insights into PBC prevention and treatment.

The Genus *Lachnospiraceae_UCG_004* group, which belongs to the clostridial cluster XIVas, is known for its 7α-dehydroxylated activity (Ridlon et al., 2016). This activity regulates SBAs by converting primary bile acids (PBAs) into SBAs through 7α-dehydroxylation (Kriaa et al., 2019). Heightened levels of this enzyme could increase SBA abundance. However, the inefficient elimination of SBAs through metabolism leads to their accumulation in bile, possibly contributing to the development of cholestasis (Li et al., 2017). Moreover, SBAs have been shown to have higher hydrophobicity and cytotoxicity than PBAs, resulting in liver cell damage (Ma et al., 2018 Sanyal et al., 2021). Observational studies by Ma et al. (2018) also found significantly higher levels of *Lachnospiraceae* in PBC patients compared to the healthy control group. These results were similar to our findings in that the *Lachnospiraceae_UCG_004* group increased the risk of PBC. However, further research is needed to elucidate the underlying mechanisms by which *Lachnospiraceae* influences PBC and explore its potential as a therapeutic target for preventing and treating this multifaceted condition.

The Order *Selenomonadales and one of its classes, Negativicutes,* belong to the *phylum Firmicutes*. Previous observational studies using 16S rRNA gene sequencing of the fecal microbiome have consistently shown a significant increase in the bacterial abundance of *Firmicutes* species in PBC patients (Ma et al., 2018). Several observational studies also showed that an increased relative abundance of *Firmicutes* is commonly associated with obesity (Mathur and Barlow, 2015; Hu et al., 2022). A recent study utilizing MR analysis provided evidence suggesting that an elevated BMI, as determined by genetic factors, could be a causal factor in the development of PBC (Xu H. et al., 2022). These studies further confirmed our results of the *Selenomonadales* group, which significantly increases the risk of PBC.

Notably, *Bifidobacterium* species are recognized as pivotal regulators of intestinal homeostasis and have the potential to confer various health benefits (Hidalgo-Cantabrana et al., 2017). Conflicting outcomes have emerged from observational studies examining their impact. A case–control study demonstrated that the relative abundance of *Bifidobacterium* showed a remarkable increase in patients with PBC compared to the control group (Furukawa et al., 2020). This finding aligns with our study and suggests a causal association between the higher prevalence of *Bifidobacterium* and increased susceptibility to PBC, highlighting its adverse impact on the disease. Furthermore, *Bifidobacterium adolescents* have been found to enhance Th-17 cell levels in diverse gut-associated organs. Increased levels of Th-17 cells have been strongly linked to autoimmune and inflammatory diseases in both mice and humans (Tan et al., 2016). Synthesizing the findings from these studies, *Bifidobacterium* significantly increases the risk of PBC, most likely through the Th-17 pathway (López et al., 2010).

Our investigation revealed a significant negative association between Family *Ruminococcaceae* and *Peptostreptococcaceae* with PBC risk, suggesting a potential protective effect against PBC. Previous research has shown that *Ruminococcaceae*, a butyrate-producing bacterial genus primarily found in the gut, is significantly reduced in PBC patients (Ohira et al., 2017; Furukawa et al., 2020). Butyrate, a short-chain fatty acid (SCFA), plays a crucial role in maintaining the integrity of the intestinal barrier. Studies have demonstrated that butyrate enhances intestinal barrier function by upregulating tight junction proteins such as claudin-1 and Zonula Occludens-1 (ZO-1) (Wang et al., 2012). Therefore, the reduced relative abundance of *Ruminococcaceae* in PBC patients may contribute to increased gut permeability. While most *Peptostreptococcaceae* bacteria are generally considered harmful, recent research has indicated that an increased population of *Peptostreptococcaceae* may protect against obesity in mice by promoting bile acid metabolism (Song et al., 2023). Nevertheless, limited research exists concerning the connection between the Family *Peptostreptococcaceae* group at the genus level and PBC. Significantly, our investigation has, for the first time, unveiled a protective causal association of the Family *Peptostreptococcaceae* group regarding PBC.

It should be noted that some specific findings in this study diverged from previous research. We attribute these disparities, at least in part, to differences in sample size, geographic background, dietary habits, and age among subjects across different studies. Fluctuations in the microbiome’s composition can lead to disturbances in its functioning and the production of abnormal metabolites. A meticulous gut microbiota analysis holds great promise in establishing a comprehensive evaluation system.

This study represents the first examination of the casual relationship between the gut microbiome and PBC risk. Through MR analysis, we have identified a causal link between the gut microbiome and PBC, with specific gut bacteria playing an active role in disease development. One notable strength of our research is the robust application of the MR method, which effectively addresses potential reverse causation and confounding factors, leading to more accurate causal inferences.

However, some limitations should be considered. Firstly, due to the limited information available in the GWAS database, the use of summary statistics for disease types rather than raw clinical data in the research made it impossible to perform additional subgroup analyses concerning disease subtypes and severity. Secondly, it is essential to acknowledge that the GWAS studies primarily included individuals of European ancestry, which may introduce biases due to variations in dominant gut bacteria influenced by diverse exposure factors like diet (Lee et al., 2018). Therefore, caution is needed when generalizing our findings to other ethnic groups. Thirdly, our study examined SNPs of gut microbiota across multiple taxonomic levels, including phylum, class, order, family, and genus, rather than a more specific species-level analysis. Although we acknowledge this limitation, it was crucial for obtaining a more comprehensive understanding of the gut microbial landscape. Furthermore, due to the complex pathobiology of PBC and the multitude of statistical complexities involved, a rigorous multiple-testing correction might inadvertently fail to identify strains causally associated with PBC. To mitigate this concern, we exercised caution and chose not to implement multiple corrections. Finally, despite our comprehensive investigation, the precise mechanisms underlying the impact of gut microbiota on the risk of PBC still require clarification and further exploration.

## 5. Conclusion

Our study revealed potential causal implications for PBC from the presence of five genera in the gut microbiome. Specifically, *Ruminococcaceae* and *Peptostreptococcaceae* were found to have a negative association with the risk of PBC. In contrast, *Selenomonadales*, *Bifidobacteriales*, and *Lachnospiraceae_UCG_004* appeared to have a potentially adverse effect on PBC. These results imply that these gut microbiota taxa may offer new opportunities for the development of treatments and preventive measures for PBC and could serve as potential biomarkers.

## Data availability statement

The original contributions presented in the study are included in the article/Supplementary material, further inquiries can be directed to the corresponding author.

## Author contributions

JZ: Writing – review & editing, Visualization, Writing – original draft. GW: Writing – review & editing, Data curation, Software. YT: Writing – review & editing, Investigation, Methodology. HL: Investigation, Writing – review & editing, Formal analysis. XG: Writing – review & editing. RP: Writing – review & editing, Data curation. JC: Writing – review & editing, Methodology. DT: Writing – review & editing, Project administration. BS: Writing – review & editing, Investigation. SJ: Writing – review & editing, Resources. GJ: Writing – review & editing, Project administration. CZ: Writing – review & editing, Funding acquisition, Resources. DB: Funding acquisition, Resources, Writing – review & editing.
