# Supplementary material for: Causal associations between gut microbiota and primary biliary cholangitis: a bidirectional two-sample Mendelian randomization study
Source: Front Microbiol. 2023 Nov 15;14:1273024. doi: 10.3389/fmicb.2023.1273024 (PMC10684913; doi:10.3389/fmicb.2023.1273024)
Supplement: Supplementary file 1 [file Data_Sheet_1.pdf]

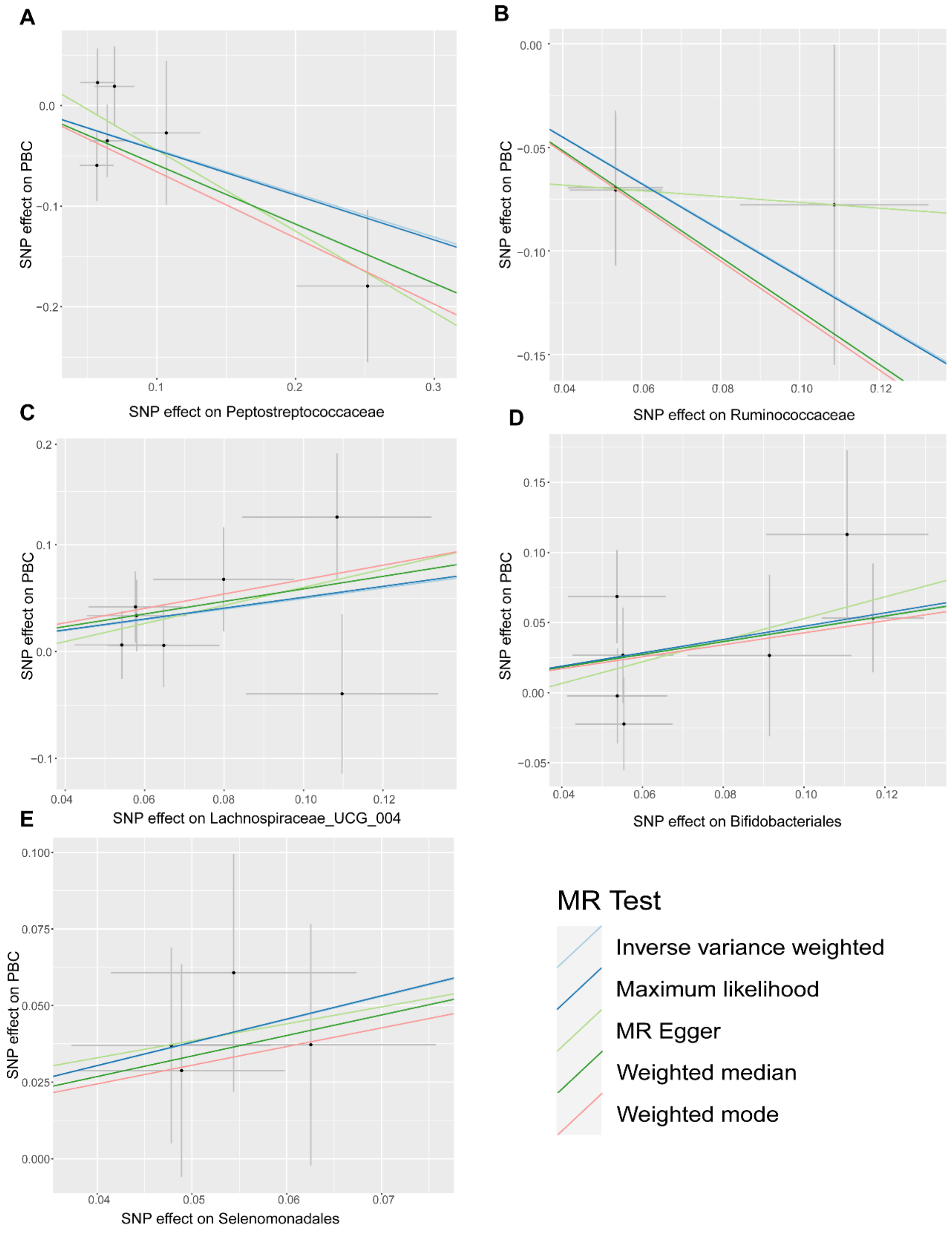

Supplementary Figure 1. Scatter plots of the MR estimates for the significant causality of five gut microbiota taxa and the risk of PBC. (A-E) The potential causal effect of five gut microbiota taxa on PBC. The lines implying positive correlations moved diagonally upward from left to right, indicating a facilitative effect of gut microbiota on PBC. The horizontal and vertical lines indicated each correlation's 95% confidence interval. The lines implying negative correlations move diagonally downward from left to right, indicating the inhibitory effect of gut microbiota on PBC. (MR, Mendelian randomization; SNPs, single nucleotide polymorphisms).

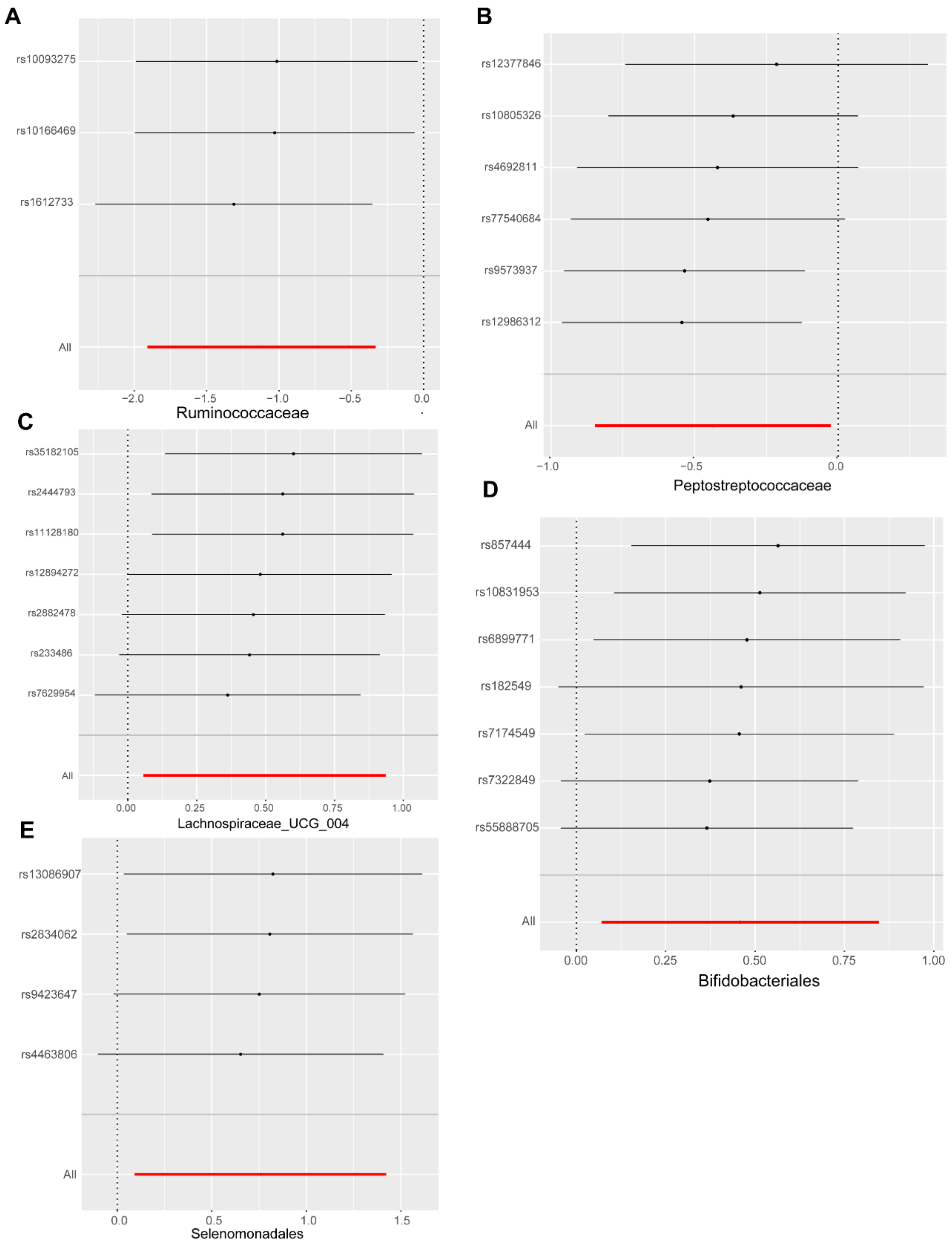

Supplementary Figure 2. Leave-one-out plots for the causal association between gut microbiota and PBC.
